# The nature and causes of unintended events reported at ten emergency departments

**DOI:** 10.1186/1471-227X-9-16

**Published:** 2009-09-18

**Authors:** Marleen Smits, Peter P Groenewegen, Danielle RM Timmermans, Gerrit van der Wal, Cordula Wagner

**Affiliations:** 1 NIVEL, Netherlands Institute for Health Services Research, Utrecht, The Netherlands; 2 Department of Sociology and Department of Human Geography, Utrecht University, Utrecht, The Netherlands; 3 Department of Public and Occupational Health, EMGO Institute for Health and Care Research, VU University Medical Center, Amsterdam, The Netherlands

## Abstract

**Background:**

Several studies on patient safety have shown that a substantial number of patients suffer from unintended harm caused by healthcare management in hospitals. Emergency departments (EDs) are challenging hospital settings with regard to patient safety. There is an increased sense of urgency to take effective countermeasures in order to improve patient safety. This can only be achieved if interventions tackle the dominant underlying causes. The objectives of our study are to examine the nature and causes of unintended events in EDs and the relationship between type of event and causal factor structure.

**Methods:**

Study at EDs of 10 hospitals in the Netherlands. The study period per ED was 8 to 14 weeks, in which staff were asked to report unintended events. Unintended events were broadly defined as all events, no matter how seemingly trivial or commonplace, that were unintended and could have harmed or did harm a patient. Reports were analysed with a Root Cause Analysis tool (PRISMA) by an experienced researcher.

**Results:**

522 unintended events were reported. Of the events 25% was related to cooperation with other departments and 20% to problems with materials/equipment. More than half of the events had consequences for the patient, most often resulting in inconvenience or suboptimal care. Most root causes were human (60%), followed by organisational (25%) and technical causes (11%). Nearly half of the root causes was external, i.e. attributable to other departments in or outside the hospital.

**Conclusion:**

Event reporting gives insight into diverse unintended events. The information on unintended events may help target research and interventions to increase patient safety. It seems worthwhile to direct interventions on the collaboration between the ED and other hospital departments.

## Background

Several studies in various countries have shown that a substantial number of patients suffer from adverse events in hospitals. [1-9] These studies have reported adverse event incidence rates ranging from 3% to 17% of all hospital admissions, with 25% to 50% of the adverse events considered preventable. The Harvard Medical Practice Study found that the third most common site of adverse events in hospitals is the emergency department (ED) and 70% of these events are due to negligence.[10] The Utah and Colorado study confirmed that adverse events in emergency medicine are highly preventable: the ED had the largest percentage of negligent adverse events (53%).[3]

The ED is a challenging hospital setting because high patient throughput, heavy dependence on services outside the ED (laboratory, radiology, consulting services etc.) and the diversity of clinical conditions presented.[11] Emergency care providers often have to work under conditions involving disrupted sleep cycles, multiple interruptions and acute time constraints, and they have to institute major medical interventions for patients with limited historical and diagnostic information.[12] Since large numbers of patients visit the ED, the incidence rate of adverse events in the ED and the large proportion that is preventable are alarming and require interventions. An increase in patient safety can only be achieved if these interventions tackle the right underlying causes.

Event reporting systems can provide valuable information for detecting patient safety issues in hospitals.[13] Generally, healthcare providers are not restricted to report only adverse events with patient harm in the reporting system. Other unintended events are considered useful sources of information as well. Unintended events are a broader group of events -including near misses-, that do not necessarily result in patient harm and occur more frequently than adverse events. Near misses are believed to share the same underlying failure factors as accidents that do reach the patient.[14] Evidence for this common cause hypothesis has been examined in a review of Wright and Van der Schaaf for the railway domain.[15]

With the present study, we want to examine the causes of various types of unintended events in the ED by analysing unintended event reports. Event reports can be helpful in capturing system defects (latent errors) and near misses that may not be detected by reviews of patient records.[16] Two earlier studies used reports to examine unintended events in the ED.[12,17] These studies had some methodological limitations. Both studies took place in only one hospital. The study period of Fordyce et al.[12] was only one week and Tighe et al.[17] used a database of event reports which contained little information on contributing factors. Moreover, they had no opportunity to interview involved healthcare providers. We tried to improve these designs by carrying out a study over a longer period in multiple centres, allowing for generalisation of the results, and by using interviews to complete the event reports.

The objectives of our study are to gain more insight into (1) the nature of unintended events in the ED, (2) the causes of unintended events in the ED and (3) the relationship between the type of event and the causal factor structure.

## Methods

### Study design and setting

From October 2006 to December 2007, an observational study was performed to examine the causes of unintended events at the emergency department (ED) of ten hospitals in the Netherlands: one university hospital, three tertiary teaching hospitals and six general hospitals. Unintended events were broadly defined as all events, no matter how seemingly trivial or commonplace, that were unintended and could have harmed or did harm a patient.[18] The study protocol was granted ethical approval by the VU University Medical Centre review board in Amsterdam.

The intake of departments was phased, because -for logistical reasons- we did not want all EDs to participate in the study simultaneously. The study period per ED was eight to fourteen weeks depending on the reporting speed. Healthcare providers (i.e. nurses, resident physicians, medical consultants) and clerks at the department were asked to report all unintended events that occurred, both when they were involved in an event and when they witnessed an event. In order to find the causes underlying the reported unintended events, the events were analysed by an experienced researcher using a Root Cause Analysis (RCA) tool called PRISMA-medical.[19,20] In addition, the unintended events were classified into one of the eight classes that we formulated after completion of the study by looking at common themes in the reported events: Materials and equipment, Diagnosis and treatment, Medication, Protocols and regulations, Incorrect data and substitutions, Collaboration with resident physicians and consultants, Collaboration with other departments and Other.

### Data collection

#### Reporting procedure

Before the start of the study, ED-staff received an oral and written instruction about the aim and procedure of the study. They had two alternatives for reporting the unintended events: a report card or report form. On the pocketsize report card, the name of the reporter, the moment in time, and a description of the event were requested. The report form was more elaborate and additionally requested the involvement of the reporter, the phase of care, place, some patient characteristics and consequences for the patient. A letter box was placed at the department to drop the report cards and forms. Our intention was to gather at least 50 reports per department to be able to dray valid causal factor profiles of each department. A minimum of fifty reports is recommended to capture the variety of possible unintended events (Prof. dr. T. van der Schaaf, personal communication). Staff were encouraged to report unintended events by a two-weekly newsletter, reminders during team meetings and appealing activities to direct staff's attention to reporting.

Once or twice a week a researcher visited the ED to collect the written reports and ask the reporters some questions about the reported events in short interviews. Each event report was followed by an interview with the reporter, mainly to get information on contributing factors. In case the reporter had used a report card, the additional information requested on the elaborate report form was also obtained during this interview.

Occasionally, questions were asked by telephone. No interviews were held with staff in other hospital departments than the ED.

#### PRISMA analysis

All unintended events were analysed with PRISMA-medical. PRISMA is a tool to analyse the root causes of a broad set of unintended events.[19,20] The corresponding taxonomy to classify the root causes, the Eindhoven Classification Model, has been accepted by the World Alliance for Patient Safety of the World Health Organization.[21,22] It is based on the system approach to human error of Reason [23,24] and the Skill-Rules-Knowledge based behaviour model of Rasmussen.[25]

**Figure 1:**
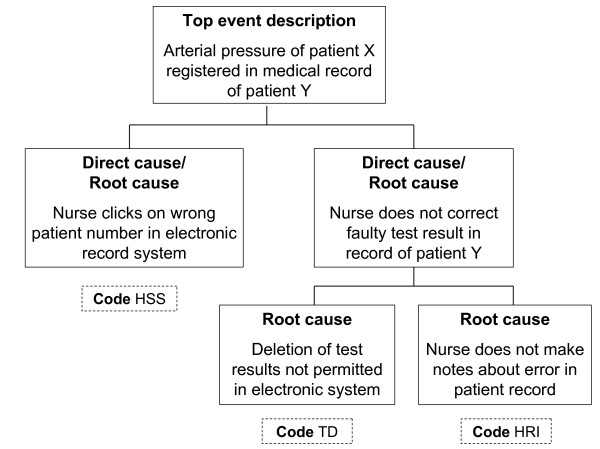
**Example of a causal tree**.

PRISMA examines the relative contributions of latent factors (technical and organisational), active failures (human) and other factors (patient related and other). Unintended events are analysed in three main steps. Firstly, a causal tree is formulated. At the top of the tree a short description of the event is placed, as the starting point for the analysis. Below the top event, all involved direct causes are mentioned. These direct causes often have their own causes. By continuing to ask "why" for each event or action, beginning with the top event, all relevant causes are revealed. In this way a structure of causes arises, until the root causes are identified at the bottom of the tree (see Figure 1). In our study, this first phase was ended when there was no more objective information of underlying causes available. Presumptions of the reporters about possible causes were not recorded in the causal tree.

Secondly, the identified root causes are classified with the Eindhoven Classification Model (ECM).[19,20,26] This taxonomy distinguishes five main categories and 20 subcategories (see Table 1).

**Table 1:** Description of categories of the Eindhoven Classification Model: PRISMA-medical version[19,20]

**Main category**		**Subcategory**	**Code**	**Description**
*Latent conditions*				
Technical		External	T-ex	Technical failures beyond the control and responsibility of the investigating organisation.
		Design	TD	Failures due to poor design of equipment, software, labels or forms.
		Construction	TC	Correct design, which was not constructed properly or was set up in inaccessible areas.
		Materials	TM	Material defects not classified under TD or TC.
Organisational		External	O-ex	Failures at an organisational level beyond the control and responsibility of the investigating organisation, such as in another department of area (address by collaborative systems).
		Transfer of knowledge	OK	Failures resulting from inadequate measures taken to ensure that situational or domain-specific knowledge or information is transferred to all new or inexperienced staff.
		Protocols	OP	Failures relating to the quality and availability of the protocols within the department (too complicated, inaccurate, unrealistic, absent, or poorly presented).
		Management priorities	OM	Internal management decisions in which safety is relegated to an inferior position when faced with conflicting demands or objectives. This is a conflict between production needs and safety. Example: decisions that are made about staffing levels.
		Culture	OC	Failures resulting from collective approach and its attendant modes of behaviour to risks in the investigating organisation.
*Active errors*				
Human		External	H-ex	Human failures originating beyond the control and responsibility of the investigating organisation. This could apply to individuals in another department.
	Knowledge-based behaviour	Knowledge-based behaviour	HKK	The inability of an individual to apply their existing knowledge to a novel situation. Example: a trained blood bank technologist who is unable to solve a complex antibody identification problem.
	Rule-based behaviour	Qualifications	HRQ	The incorrect fit between an individuals training or education and a particular task. Example: expecting a technician to solve the same type of difficult problems as a technologists.
		Coordination	HRC	A lack of task coordination within a healthcare team in an organisation. Example: an essential task not being performed because everyone thought that someone else had completed the task.
		Verification	HRV	The correct and complete assessment of a situation including related conditions of the patient and materials to be used before starting the intervention. Example: failure to correctly identify a patient by checking the wristband.
		Intervention	HRI	Failures that result from faulty task planning and execution. Example: washing red cells by the same protocol as platelets.
		Monitoring	HRM	Monitoring a process or patient status. Example: a trained technologist operating an automated instrument and not realising that a pipette dispenses reagents is clogged.
	Skill-based behaviour	Slips	HSS	Failures in performance of highly developed skills. Example: a technologist adding drops of reagents to a row of test tubes and than missing the tube or a computer entry error.
		Tripping	HST	Failures in whole body movements. These errors are often referred to as " slipping, tripping, or falling". Examples: a blood bag slipping out of one' s hands and breaking or tripping over a loose tile on the floor.
*Other factors*				
Patient related		Patient related factor	PRF	Failures related to patient characteristics or conditions, which are beyond the control of staff and influence treatment.
Other		Unclassifiable	X	Failures that cannot be classified in any other category.

Eventually, by aggregating the classifications of root causes of at least 50 events, a so called PRISMA profile can be delineated, which shows in a graphical representation the relative contributions of the different root causes and gives direction to the development of preventive strategies.[19,20]

The analyst of the unintended events in the ED was a PhD candidate on patient safety in hospitals and also one of the authors of this paper (MS). She was trained in the PRISMA method. In a previous paper, we examined the inter-rater reliability of formulating root causes in causal trees and classifying the root causes with the ECM.[27] The reliability analyses were performed with a sample of event reports from a larger database of events than used for the current study. Next to the current ED-reports, this database also contained reports from surgery and internal medicine departments. The agreement in formulating root causes of unintended events, expressed as a mean score between 0 and 3, was good (2.0). The inter-rater reliability for the number of root causes used in the causal tree, was moderate (κ = 0.45). The inter-rater reliability of classifying root causes with the ECM taxonomy was substantial at main category level (κ = 0.70) and subcategory level (complete taxonomy) (κ = 0.63).

### Statistical analysis

The data of the reports were first summarised using descriptive statistics and frequency tables. All analyses were performed with 522 cases (N = 522 unintended events), except for the analysis of the relative frequencies of causes per event type. The frequencies per event type were calculated using the 845 root causes as cases (N = 845 root causes), because we wanted the percentages in the bars to sum up to 100% to increase the comprehensibility of the figure. SPSS 14.0 was used to perform the statistical analyses.

## Results

### Characteristics of reported unintended events

The total number of events reported was 522, ranging from 46 to 71 per ED, with an average of 52 reports (SD = 7.6). In total, there were 743 reporting days during which 189 different employees reported one or more unintended events. Most reports were made by nurses (85%). Resident physicians or consultants reported 13% of the unintended events and clerical staff reported 2%. In 83% of the unintended events, the reporter was directly involved in the event.

In Table 2, a number of clinical characteristics of the unintended events are listed. Most events (44%) were known to have occurred during daytime hours and 34% during evening and night. For 22% of the unintended events, the reporter did not specify or know at what time the event occurred. The phase in ED care in which most events occurred was medical examinations/tests (36%). More than half of the unintended events (56%) had consequences for the patient. In 45% of these events with consequences for patients, the patient suffered some inconvenience, for example prolonged waiting time. In 30% the patient received suboptimal care, for example a delay in starting antibiotics treatment. For smaller groups of patients the outcomes were more severe, e.g. extra intervention (8%), pain (6%), physical injury (3%).

**Table 2:** Clinical characteristics of unintended events

**Characteristic unintended event**	**No. of unintended events (%)**
**Time **(N = 522)	
Daytime (7 am to 5 pm)	227 (43.5)
Evening and night (5 pm to 7 am)	178 (34.1)
Unknown	117 (22.4)
Phase of care* (N = 522)	
Medical examination/tests	186 (36.1)
ED stay general	84 (16.3)
Medication	48 (9.3)
Treatment/intervention	35 (6.8)
Transfer of patient	33 (6.4)
Acute situation	26 (5.0)
Hospitalisation	25 (4.9)
Discharge	12 (2.3)
Handover	9 (1.7)
Triage	6 (1.2)
Other	84 (16.3)
**Consequences for patient **(N = 522)	
Yes (see 'outcome')	294 (56.3)
No	211 (40.4)
Unknown	17 (3.3)
Outcome* (N = 294)^†^	
Inconvenience e.g. long waiting time; high temperature in ED; patient mistakenly sent to another hospital	134 (45.1)
Suboptimal care e.g. delay in starting antibiotics treatment; no surveillance of sick patient; patient mistakenly sent home	90 (30.3)
Extra intervention e.g. extra blood withdrawal, unnecessary X-ray	25 (8.4)
Pain e.g. no pain medication; missed bone fracture; no sling given after stitching of thumb wound.	19 (6.4)
Physical injury e.g. decubitus ulcer; eyelid glued	10 (3.4)
Mental injury e.g. wrong patient called back to hospital for positive test result; relatives informed about HIV of patient without patient's consent	6 (2.0)
Longer stay (> 24 hrs) e.g. admission to a nursing department	2 (0.7)
Unknown	20 (6.7)

* For a number of unintended events, more than one category was selected.

^† ^Only the groups of patients that experienced consequences of the unintended event.

Table 3 shows the types of events that were reported with some examples. A quarter of the unintended events was related to the cooperation with other departments, e.g. with laboratories and nursing wards. In 20% of the unintended events, there were problems with materials or equipment. Furthermore, relatively large parts of unintended events were related to the collaboration with resident physicians and consultants (17%) or to diagnosis and treatment (14%).

**Table 3:** Types of unintended events

**Unintended event type**	**No. of unintended events (%)** (N = 522)
Collaboration with other departments	128 (24.5)
e.g. long waiting time for laboratory test results	
e.g. not al requested X-rays made at radiology department	
e.g. difficulties finding a place at a nursing ward for the patient	
Materials and equipment	106 (20.3)
e.g. ear thermometer gives inaccurate measurements	
e.g. error in electronic record system (unable to look up medical history of patient)	
e.g. materials lacking for treatment of patient	
Collaboration with resident physicians and consultants	89 (17.0)
e.g. long waiting time for resident or consultant to come	
e.g. insufficient supervision of resident physicians	
e.g. not able to reach resident or consultant	
Diagnosis and treatment	75 (14.4)
e.g. no plaster bandage applied after fracture reposition	
e.g. eyelid glued when gluing nose bridge	
e.g. elbow injury overlooked	
Incorrect data and substitutions	39 (7.5)
e.g. incorrect date on X-ray	
e.g. appointment form given to wrong patient	
e.g. sticker with personal information of wrong patient pasted on laboratory request form	
Medication	38 (7.3)
e.g. prescription of medicine in incorrect dose	
e.g. medication expired	
e.g. medication instruction accomplished twice	
Protocols and regulations	20 (3.8)
e.g. inconsistency in protocols	
e.g. protocol untraceable on the intranet	
e.g. staff not familiar with procedure in new protocol	
Other	27 (5.2)
e.g. inadequate transport of patient	
e.g. dangerous ground sill at entrance of ED	
e.g. patient leaves hospital without being discharged	

### Causes of unintended events

All 522 unintended events were analysed with PRISMA, resulting in 845 root causes. Fifty percent of the unintended events had one root cause, 39% had two root causes, 10% three root causes and 1% four root causes. The mean number of root causes per unintended event was 1.62 (SD = 0.71).

**Figure 2:**
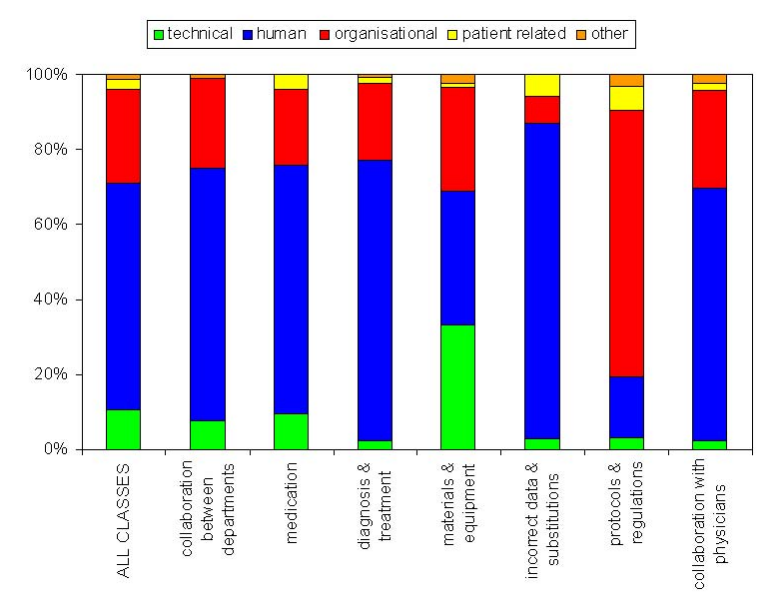
**Distribution of main causal factor groups per unintended event type (N = 845)**.

In Figure 2, the distributions of the five main groups of root causes per event type are shown. Overall, most root causes were human (60%), followed by organisational (25%) and technical (11%) root causes. Unintended events related to materials and equipment were relatively often caused by technical factors. Incorrect data and substitutions were caused for a large part by human errors, while organisational factors contributed most to unintended events related to protocols and regulations.

Table 4 shows the frequencies of the causes on subcategory level (see also Table 1 for explanation of the ECM categories). Material defects (TM) were the most common technical factors (38% of unintended events with technical causes). External factors were largely present, especially human and organisational external factors (H-ex and O-ex). These are causes originating in another department outside the ED, e.g. the laboratory or radiology. Of all 845 root causes, 387 (46%) were external. In 69% of the unintended events with human causes, an external human factor contributed to the event, for example: the surgeon on duty was in the operating room and forgot to pass the beeper to a fellow surgeon, or a laboratory worker forgot to insert a patient's test results in the computer. In 58% of the unintended events with organisational causes, there was an external organisational factor, for example a laboratory worker saved blood pipes until the testing machine was full or a hospital admission stop was ignored by a medical consultant.

**Table 4:** Causes of unintended events at the emergency department

**Main category**		Subcategory*	**Code**	**Frequency (%)^†^**	**Percentage within each main category** **(column %)^†^**
**Technical** (N = 88)		External	T-ex	19 (2%)	22%
		Design	TD	26 (3%)	30%
		Construction	TC	13 (2%)	15%
		Materials	TM	33 (4%)	38%
					
**Human** (N = 375)		External	H-ex	260 (31%)	69%
	Knowledge based	Knowledge	HKK	30 (4%)	8%
	Rule based	Qualifications	HRQ	16 (2%)	4%
		Coordination	HRC	34 (4%)	9%
		Verification	HRV	52 (6%)	14%
		Intervention	HRI	82 (10%)	22%
		Monitoring	HRM	28 (3%)	7%
	Skill based	Slips	HSS	5 (1%)	1%
		Tripping	HST	3 (0%)	1%
					
**Organisational** (N = 186)		External	O-ex	108 (13%)	58%
		Protocols	OP	35 (4%)	19%
		Transfer of knowledge	OK	17 (2%)	9%
		Management priorities	OM	32 (4%)	17%
		Culture	OC	19 (2%)	10%
					
**Patient related** (N = 20)		Patient related	PRF	20 (2%)	100%
**Other** (N = 13)		Other	X	13 (2%)	100%
*Total*				845 (100%)	

* See Table 1 for description of categories.

^† ^More than one causal factor per event possible.

When looking at the internal causes inside the ED, human intervention errors (HRI) stand out (22% of unintended events with human causes). Examples of intervention errors are: not recording the time when medication was administered or not plugging the battery of a medical device in the socket. Moreover, errors with regard to protocols (OP) and management priorities (OM) are relatively frequent organisational causes (respectively 19% and 17% of all unintended events with organisational causes), for example protocols that were incomplete or old medical devices that were not replaced by hospital management.

## Discussion

### General findings and interpretation

We gathered and analysed a large number of unintended events (522) using a root cause analysis tool based on the sound theoretical frameworks of Reason and Rasmussen, which is accepted by the WHO and which has a good reliability.[27]

The results show that a large number of unintended events occur in the collaboration with departments outside the ED (laboratory, radiology, consulting services etc). Staff in the ED are heavily dependent on these services. The problems in the cooperation with outside services can also be noticed in the phase of care in which unintended events mainly come about -medical examinations and tests-, since a lot of tests are performed in other departments.

Half of all reported events reached the patient directly, most often resulting in inconvenience or suboptimal care. The causes of the unintended events were mainly human, though system factors (organisational and technical) were established as well. Predominance by human causes is also found in the aviation industry. It is estimated that approximately 75 percent of all aviation accidents are related to human errors.[28] Nearly half of all causes we found were external, meaning that an individual's behaviour, technical factors or organisational factors at an outside department contributed to the unintended event. This also confirms the finding that there are problems in the cooperation with other departments, although we have to bear in mind that people feel less constrained reporting unintended events originating in other departments than in their own.

Unintended events related to materials and equipment were relatively often caused by technical factors. Incorrect data and substitutions were for a relatively large part caused by human errors, while organisational factors contributed most to unintended events related to protocols and regulations.

Some comments have to be made for a good interpretation of the causes of the unintended events. Firstly, the reported unintended events were related to patient care, and healthcare providers were somehow involved in all events. This resulted in involvement of human causes in many cases. The PRISMA analysis, however, did focus on identifying accompanying system factors, beside these human causes. Secondly, as we strived for objective information about underlying causes, presumptions of the reporters about possible organisational or technical causes were not recorded in the causal tree. Finally, a lack of organisational or technical barriers was not labeled as an organisational or technical cause. An example: when two healthcare providers make the same laboratory request for a patient, blood is taken unnecessarily once. An automatic electronic signaling system would have prevented the double laboratory request, but as long as such a system does not exist at the ED, this system can not be regarded as a cause. Yet, improvements of organisational procedures or technique can arise from the identification of human errors.

### Limitations

The unintended events identified in our study are unlikely to be a random sample of all unintended events occurring in the ED, whereas not all unintended events that took place will have been reported. Since the healthcare providers making the reports were often directly involved in the patients' care and since the reporting was not anonymous, it is possible that certain mistakes were under-reported because they were embarrassed or afraid of condemnation by the researchers or colleagues. This may have biased the results towards the reporting of less significant events, events without consequences for the patient and errors originating in other departments, because these are 'safer' to report. Anonymous reporting would perhaps have yielded more events, but interviewing the reporters -essential for obtaining information on contributing factors- would not have been possible in that case. Some unintended events occurred multiple times at one ED, and some healthcare providers informed us not to be willing to report these events over and over again. Examples are long waiting times for laboratory test results or for (paper) patient records from the records archive. We do not know exactly which events were under-reported, how frequently they occurred and whether they had the same underlying causes in every case. Therefore, we were not able to correct for this under-reporting by giving different weights to these types of events and their causes. Finally, most unintended events were reported by nurses. Consequently, the study mainly gives an idea about events related to nursing care and to a lesser extent to care processes by residents and specialists in the ED.

Another limitation may have had an effect on the root causes identified. The interviews about the events depend on the recall of the reporter. However, we strived for a small time lag between the occurrence of the event and the interview. Events were discussed within a few days, with a maximum time lag of three weeks in some exceptional cases.

### Comparison with previous studies

As we mentioned in the introduction, two other event reporting studies have been performed in hospital EDs in the past. Fordyce et al.[12] examined 346 error reports. The area of emergency care in which most events occurred was 'diagnostic studies'. In their study of 174 event reports, Tighe et al.[17] found that the largest category of events concerned delays, for example difficulties in arranging for a patient to be seen promptly by a medical specialist. These findings correspond to our results, as the most frequently reported unintended events in our study concerned the collaboration with services outside the ED performing diagnostic tests and the collaboration with medical consultants, mainly resulting in delays for the patient. Another large group of events -problems with materials and equipment- was not found in the other two studies.

In both studies, as well as in our study, only small numbers of events had severe consequences for the patient: Fordyce et al.[12] found adverse outcomes in 2% of the reports and in the study of Tighe et al.[17], approximately 11% of the reported events were classified as 'serious'. However, we cannot compare the causes identified in our study with these previous studies. Fordyce et al.[12] did not investigate causes of errors and Tighe et al.[17] stated that the reports in their database did not include enough information on contributing factors.

### Implications for practice

We recommend improving the collaboration between the ED and other hospital departments, while a large number of unintended events occur in the collaboration with departments outside the ED and nearly half of all causes were external. A reduction of the external factors is not only the responsibility of these external departments. We believe that EDs and other departments should jointly discuss these causes and work on improvement plans for safe patient care across hospital departments (e.g. improving communication during consultations of medical specialists and agreements with laboratory about the processing of lab requests).

Causes of unintended events were predominantly labelled as human. In 2008, the Dutch Society of Medical Specialists, among others, has formulated a national patient safety action campaign for hospitals 'Prevent harm, work safely' that contains interventions directed at reducing human error. Elements of the programme are: education about patient safety, team training and evaluations of the Individual Functioning of Medical Specialists (IFMS), including the construction of a personal portfolio, a personal progress plan and annual interviews about quality of care and communication with colleagues and patients.[29] These interventions might be valuable for hospitals, and more specifically EDs, in other countries too. However, improvement efforts should not be solely directed at the behaviour of healthcare personnel. Many of the unintended events were caused by a combination of latent factors (organisational or technical) and active (human) factors. We therefore recommend interventions to be aimed at the system that surrounds healthcare professionals. Great gains in safety can be achieved through relatively small modifications of equipment and workplaces [30,31]. Examples are a decrease in the variability of procedures or the design of devices which reduces mental workload and decision-making (e.g. a single telephone number across the country for calling resuscitation teams or colour coding for alerts on patient wristbands)[31] and building in barriers in the system when an error is made (e.g. a computer signal in case of a contraindication).

Finally, we believe that event reporting and analysis gives valuable insight into the nature and causes of unintended events. The PRISMA method to analyse events is a useful tool to examine large numbers of events to uncover latent and active causal factors. It can be taught to and used by researchers, clinicians and managers. In the Netherlands, many hospitals start using PRISMA to study events reported by their staff. Most hospitals are taking on decentralised (department-level) event reporting with in each department a special committee that has the task to analyse the reported events, give staff feedback and design and implement improvements.

### Recommendations for future research

While in most unintended events in our study no harm for the patient was involved, only a small number of the unintended events would have met the criteria of an adverse event: 1) an unintended (physical and/or mental) injury which 2) results in temporary or permanent disability, death or prolongation of hospital stay, and is 3) caused by health care management rather than the patient's disease. The events in our study were not assessed by physician reviewers on these criteria. It is unclear whether the results regarding the causes of the broad group of unintended events we examined are also applicable to the specific group of adverse events. Although the common cause hypothesis of near misses and accidents is supported by research in the railway sector[15], future research is needed to examine the resemblance of the causal factor structures of unintended events and adverse events in the healthcare domain.

Our study mainly gives an idea about events related to nursing care. To get a more complete view of all unintended events that occur, we recommend expanding the reporting of events with patient record review. The report of Wagner et al.[32] showed that there was almost no overlap in the events reported by staff and the events identified trough patient record review. The unintended events identified in patient records were more often related to medical care by physicians, than the events that were reported by staff. Record review can be considered as an important additional source to voluntary reporting of unintended events, primarily to find more unintended events related to physician/specialist care.

## Conclusion

Our study shows that event reporting gives insight into diverse unintended events that occur within healthcare, especially nursing care. The majority of unintended events had no consequences for the patient or resulted only in minor patient inconvenience. However, since large numbers of patients visit the ED, the accumulated effect of the events on patient well-being and the healthcare delivery system is likely to be large.

The information on unintended events may help target research and interventions to increase patient safety. It seems worthwhile to direct interventions on the collaboration between the ED and other hospital departments, because a large number of unintended events occur in the collaboration with departments outside the ED and nearly half of all causes were external.

The causes of the unintended events were mainly human, though since latent factors -organisational and technical- were established as well, and believing that various human causes can be captured by building organisational and technical defences, we recommend to explore effective system interventions to improve patient safety in the emergency setting.

## Competing interests

The authors declares that they have no competing interests.

## Authors' contributions

GW and CW obtained research funding. MS, CW and DT were involved in the design of the study. MS coordinated the data collection, analysed the events and wrote the manuscript. MS and CW performed the statistical analyses of the data. CW, PG, DT and GW were involved in revising the manuscript critically for important intellectual content. All authors read and approved the final manuscript.

## Pre-publication history

The pre-publication history for this paper can be accessed here:


